# Perspective on the clinical management of post-stroke aphasia and dysphagia using repetitive transcranial magnetic stimulation (rTMS)

**DOI:** 10.3389/fneur.2024.1417641

**Published:** 2024-06-27

**Authors:** Anastasios M. Georgiou

**Affiliations:** ^1^The Cyprus Rehabilitating Aphasia and Dysphagia (C-RAD) Lab, Department of Rehabilitation Sciences, Cyprus University of Technology, Limassol, Cyprus; ^2^The Brain and Neurorehabilitation Lab, Department of Rehabilitation Sciences, Cyprus University of Technology, Limassol, Cyprus

**Keywords:** stroke, aphasia, neurostimulation, TMS, methodological rigor

## 1 Introduction

Stroke, a prevalent cause of disability worldwide, presents individuals with a range of formidable challenges; notably aphasia, reported at rates of 7%−77% across high- and middle-income countries (1), and dysphagia, with incidence rates reaching up to 80% (2). Despite their distinct manifestations, these conditions are pivotal for survival, human communication, and overall quality of life (QoL) post-stroke. Within the domain of medical speech pathology, aphasia and dysphagia have historically been key areas of therapeutic intervention, primarily through behavioral therapies. However, the advent of technological advancements has fueled interest in exploring noninvasive brain stimulation techniques (NIBST), such as repetitive transcranial magnetic stimulation (rTMS), for their potential in leveraging neuroplasticity to address these post-stroke deficits.

## 2 Repetitive TMS applications in stroke-induced aphasia and dysphagia

Since the 1990s, rTMS has attracted attention as a promising therapy for treating problems associated with various neurological disorders, notably including aphasia and dysphagia post-stroke. This interest is driven by the safety and non-invasive nature of rTMS, with ongoing research uncovering new treatment options. Several factors, as analyzed below, are intricately related to the clinical effects of rTMS in the treatment of stroke induced aphasia and dysphagia.

### 2.1 The role of brain laterality

For over a century after Broca's and Wernicke's first reports, language has been hypothesized to be lateralized to the inferior frontal and superior temporal areas of the left hemisphere in adults. Building on this understanding, research applying rTMS as a treatment for aphasia following stroke commonly adopts one of two strategies that are principally informed by models of post-stroke brain reorganization. The first strategy involves down-regulating neuronal activity in right hemisphere regions, typically by targeting contralateral areas homologous to the lesion, using inhibitory stimulation protocols that apply low-frequency rTMS [e.g., (3–5)]. The second aims to increase activity in perilesional areas of the left hemisphere using excitatory forms of stimulation that employ high-frequency rTMS [e.g., (6, 7)]. Some scientists have used a combined approach involving both suppressing activity in the right hemisphere and up-regulating the left hemisphere utilizing bilateral rTMS [e.g., (8, 9)]. In any case, it is well established that language processes rely on bilaterally distributed brain networks and therefore the role of the right hemisphere is neither disregarded nor overlooked. For instance, while the lesion size in the left hemisphere is the most significant predictor of stroke induced aphasia recovery after 6 months, the volume of the long segment of the right arcuate fasciculus is a good predictor of longitudinal recovery as well (10). Hence, suppression of neuronal activity in the right hemisphere targets the hyperactive right pars triangularis (pTr), which works maladaptively for recovery, in order to facilitate modulation of the right pars opercularis (pOp) that in turn promotes language recovery via secondary pathways [see (11) for details].

While the concept of brain laterality is widely accepted within the aphasia research community, the cerebral control of swallowing presents a more debated topic among dysphagia researchers. Cumulative research findings suggest that both reflexive and volitional swallowing are regulated by various cortical and subcortical regions (i.e., primary motor cortex, primary somatosensory cortex, insula, cingulate cortex, supplementary motor area, premotor cortex, auditory cortex, inferior frontal gyrus, parietooccipital and prefrontal cortex, operculum, putamen, thalamus, global pallidus, internal capsule, cerebellum, corpus callosum, basal ganglia, caudate, pons, midbrain and inferior parietal lobule) and damage to these areas can lead to dysphagia. These areas are interconnected in distinct groups within and between both hemispheres - for a comprehensive review on the topic, refer to Cheng et al. (12) and the references within. Even though several human studies indicate hemispheric dominance in swallowing, either favoring the left [e.g., (13–15)] or the right [e.g., (16, 17)], the degree of laterality varies among individuals with activation patterns and laterality shifting between preparation and execution stages (12). Additionally, it remains unclear if dysphagia symptoms are more likely to arise from lesions in the left or right hemisphere. Some studies find no link between the lesioned hemisphere and dysphagia severity, while others suggest more severe dysphagia with either right of left hemispheric lesions (12). Given these complexities and refraining from necessarily viewing the right hemisphere as maladaptive for swallowing improvement post-stroke, as posited by the interhemispheric inhibition model (18), both excitatory and inhibitory rTMS paradigms have been applied unilaterally to either the left or right hemispheres, or bilateral stimulation has been utilized [e.g., (19–27)]. It is important to note though that several studies have not reported favorable outcomes with rTMS for post-stroke swallowing rehabilitation (28–31). So, maybe it is insufficient to solely take into consideration the various nodes of swallowing and lesion location. Of particular interest are the key areas influencing long-term recovery from post-stroke dysphagia. Evidence suggests that in ischemic stroke, the risk of aspiration is more likely to persist beyond the first week when both the frontal operculum and insular cortex are affected (32). Furthermore, damage to the insular cortex may impair ipsilesional cortical reorganization and slow recovery, with lesions affecting more than 50% of that node linked to impaired oral intake after 4 weeks and those affecting < 25% of it associated with recovery (33). Given this, rTMS can be strategically used to target the insular cortex, potentially enhancing cortical reorganization, and accelerating the recovery of swallowing function in stroke patients.

Recently, researchers have begun to investigate the potential of the cerebellum to facilitate recovery for post-stroke aphasia and dysphagia using rTMS. Specifically, low-frequency rTMS has been explored for post-stroke aphasia (34), while three studies (35–37) have applied high-frequency rTMS to the cerebellum for dysphagia, yielding favorable outcomes. These findings suggest that rTMS targeting the cerebellum holds promise for promoting recovery in both conditions. With regards to aphasia, it is assumed that neuromodulation of the right cerebellum regulates functional connectivity between the right cerebellum and the cerebral areas involved in language processing thereby facilitating language gains. With regards to dysphagia, the physiologic mechanism of action involves the cerebellum indirectly modulating neuronal activity not only in the brainstem but also in other brain areas involved in swallowing, such as the insular cortex and cerebrum.

Nonetheless, there is currently no consensus in relation to the optimal stimulation site (i.e., affected, unaffected, or both hemispheres, or cerebellum) and precise stimulation parameters for dysphagia and aphasia post-stroke. This lack of agreement highlights the urgent need for additional research to find the most effective rTMS approaches for treating these conditions. Additionally, standardization of stimulation protocols and rigorous investigation into individual patient characteristics may lead to the development of personalized treatment strategies.

### 2.2 Importance of baseline clinical, neuroradiologic and instrumental assessment

Baseline clinical and neuroradiologic assessments for aphasia are essential components for the successful application of rTMS in post-stroke rehabilitation. Comprehensive baseline assessments enable the (i) determination of the nature and extent of aphasia, (ii) recognition of potential risks and (iii) possible forecasting of recovery trajectories. Such assessments also guide more precise and effective intervention strategies tailored to individual patient needs. Precise information on the syndrome (e.g., global vs. motor vs. sensory aphasia) and its severity is crucial because these factors are associated with different prognoses. Recovery from aphasia is a dynamic process, and it is often observed that one type of aphasia evolves into another (38, 39). For instance, global aphasia typically evolves into Broca's aphasia (40), indicating that rTMS protocols may preferably focus initially on broader language functions before targeting speech production. Similarly, patients with vascular etiology presenting with Broca's aphasia, which often improves to anomic aphasia (40), may benefit from rTMS protocols designed to progressively enhance fluency and word retrieval. For patients with vascular etiology presenting with Wernicke's aphasia, who show potential for comprehension improvement and evolution to conduction or transcortical sensory aphasia (40), rTMS may focus on enhancing auditory comprehension, semantic processing, and naming. Understanding these prognostic nuances would allow clinicians to tailor rTMS protocols to the specific type of aphasia and its likely progression, optimizing rehabilitation outcomes for post-stroke patients. Such considerations are currently lacking in the existing literature.

Baseline clinical, neuroradiologic, and instrumental assessment for post-stroke dysphagia is also very important when considering the application of rTMS for rehabilitation purposes. Such assessments serve as the foundation for understanding the underlying physiological and neurological mechanisms contributing to stroke induced dysphagia. Clinical assessment provides valuable information on the patient's swallowing function, including the severity of dysphagia and associated symptoms. Neuroradiologic examinations help with the identification of structural abnormalities or lesions in the brain that may be contributing to swallowing difficulties, offering insights into the neuroanatomical basis of dysphagia. Instrumental assessments, such as videofluoroscopy (VFS) or fiberoptic endoscopic examination of swallowing (FEES), allow for real-time visualization of the swallowing process, enabling clinicians to identify specific impairments and tailor interventions accordingly. By conducting comprehensive assessments across these domains, clinicians can gain a holistic understanding of the patient's dysphagia profile, thereby informing the development of targeted rTMS treatment protocols aimed at addressing the underlying neural mechanisms and improving swallowing function.

### 2.3 Measuring and control in rTMS studies: aphasia vs. dysphagia post-stroke

In studying dysphagia and aphasia, researchers encounter unique challenges in measuring and controlling variables. Dysphagia research benefits from a more straightforward measurement process due to the universal nature of swallowing as a physiological function. This universality enables consistent assessment and interpretation of treatment outcomes across patients from different cultural backgrounds. On the other hand, aphasia studies face complexities in terms of measuring variables due to the diverse linguistic and cultural contexts in which they are conducted. In aphasia research standardizing assessment tools and outcome measures across various linguistic contexts is very important as it allows for meaningful comparisons across studies and ensures the validity and reliability of research findings.

Moreover, in comparison to dysphagia trials, controlling factors in aphasia studies, such as the implementation of behavioral interventions, introduces additional challenges. Behavioral therapies for aphasia involve a diverse range of approaches, with varying regimens applied across different studies. With regards to speech and language therapy (SALT) that is used as an adjuvant therapy in rTMS trials, significant inconsistencies in SALT types and intensities are observed across studies and such inconsistencies can impact the interpretation of results and the ability to make meaningful comparisons across studies. For instance, one relevant study focused on language comprehension and expression with a 30-min program (41), another trial implemented a 30- min SALT program focusing on naming (42) and another research utilized a 45-min SALT regimen targeting the reactivation of word retrieval (3). Other studies have implemented a 45-min SALT regimen tailored to address patient-specific language difficulties [e.g., (43–45)]. The lack of SALT standardization across studies hampers the assessment of rTMS efficacy, making it difficult to discern the specific outcomes of rTMS from those of SALT. As a result, the true extent of improvement in language abilities ascribed to rTMS remains uncertain. On the other hand, dysphagia studies experience fewer differences in controlling factors, as interventions often focus on physiological aspects of swallowing that are less influenced by cultural and/or linguistic factors. Overall, while dysphagia research benefits from a more streamlined tracking process and simpler control factors, aphasia studies entail greater complexity due to linguistic and cultural diversity, especially when considering bilingual or multilingual stroke patients.

The possibility that rTMS may prime the brain to receive behavioral aphasia and dysphagia therapy raises interesting questions about the possible synergistic effects of combining these interventions. Repetitive TMS has demonstrated neuromodulatory effects on cortical excitability and neural plasticity, which, theoretically, can enhance the brain's responsiveness to subsequent interventions. Nevertheless, the extent to which rTMS primes the brain for behavioral treatment, particularly in the context of post-stroke aphasia and dysphagia, remains an active area of research. With regards to post-stroke aphasia, recent studies indicate that when rTMS is used as a standalone treatment, it holds promise in facilitating language and/or cognitive gains [e.g., (5, 46–48)]. With regards to stroke-induced dysphagia recent (MAs) analyses have indicated mixed outcomes [e.g., (49–52)]. Therefore, the question arises: is behavioral therapy indispensable, or can it be substituted by rTMS treatments? Well, while rTMS has the capacity to modulate neuronal activity directly and indirectly, potentially accelerating neuroplasticity and recovery, behavioral therapy provides essential benefits that rTMS alone cannot fully induce, such as personalized strategies for daily functioning and psychosocial support. Thus, an integrated approach that combines both behavioral therapy and rTMS might offer the most comprehensive and effective treatment for stroke induced aphasia and dysphagia. Additional research is required to comprehensively understand the potential of rTMS as a standalone therapy and to determine the best protocols for its use in conjunction with behavioral treatments.

### 2.4 Assessing the merits of meta-analyses

A notable shortcoming of both systematic review (SRs) and meta-analyses (MAs) is that their conclusions can vary significantly with the inclusion or exclusion of certain studies. Also, the credibility of this type of research relies on the quality of the studies SRs and MAs include, as biased studies can exacerbate overall bias and therefore lead to misleading conclusions (53). In the field of rTMS for post-stroke aphasia and dysphagia, there is currently a noticeable trend among researchers to generate multiple MAs, surpassing the number of available primary studies. While MAs are valuable tools for synthesizing existing evidence and offering insights into intervention effectiveness, the disproportionate increase in MAs, in comparison to primary studies, raises concerns regarding the reliability and robustness of their findings. This surge in the number of MAs leads to redundancy and a decrease in the overall quality of research in these areas and therefore highlights the importance of maintaining a balance between conducting MAs and primary studies.

Umbrella reviews (URs) enhance the objectivity of SRs by offering a comprehensive overview and quality control. They also streamline research and foster collaboration, thus reducing redundancy and improving research quality amidst the proliferation of MAs. A recent UR on the effectiveness of rTMS for dysphagia in stroke patients (54) revealed significant overlap among studies included in various MAs, which is unsurprising given the disproportionately larger number of MAs compared to primary studies on the topic. Repeatedly including the same studies in MAs consolidates evidence and strengthens statistical power, yet it may also reinforce specific result patterns, emphasize potentially misleading outcomes, and result in an overly precise yet inaccurate estimation of intervention effectiveness. This issue becomes particularly worrisome when SRs receive low ratings in methodological quality, as it casts doubt on the reliability of conclusions derived from the MAs contained within these SRs. This was the case for two umbrella reviews, one for rTMS post-stroke aphasia (55) the other one for rTMS post-stroke dysphagia (54). Both URs found that published SRs, with or without MAs, of randomized controlled trials (RCTs) on the topics exhibit subpar methodological quality and therefore the evidence concerning the effectiveness of rTMS in promoting language and swallowing improvements post-stroke is inconclusive.

To address challenges associated with SRs and MAs, researchers should prioritize methodological rigor in their execution. Specifically, efforts to enhance the quality of SRs should align with established guidelines, alongside fostering collaboration among researchers to avoid duplication of efforts and ensure that the synthesis of evidence is comprehensive and reliable.

### 2.5 The importance of dissociating clinical from statistical significance

The distinction between clinical and statistical significance is very important in rTMS research for post-stroke aphasia and dysphagia. While statistical significance is very important for researchers, clinical significance is more important for individuals with aphasia or dysphagia post-stroke and their caregivers. Despite achieving statistical significance in treatment outcomes, the failure to accommodate the needs and fulfill the expectations of participants and their caregivers underscores the importance of measuring clinical significance. Quality of life encompasses a wide range of aspects, including physical health, mental health, emotional wellbeing, and the ability to engage in meaningful activities. For patients recovering from stroke, improvements in these areas can be just as important as neurological recovery. Therefore, incorporating QoL measures tailored to post-stroke aphasia and dysphagia would allow for a comprehensive understanding of treatment effectiveness and its implications on patients' lives. Despite the significance of QoL measures, they are seldom utilized in rTMS studies addressing these conditions. To advance research in those fields, it is imperative to integrate ecological outcome measures that reflect the real-world impact of interventions, facilitating a more holistic evaluation of treatment effectiveness and patient outcomes.

## 3 Discussion

Despite ongoing and increasing research on rTMS in stroke-induced aphasia and dysphagia, as of 2020, Lefaucheur et al. (56) concluded in their extensive review of rTMS studies up to 2018 that the evidence supporting rTMS effects on post-stroke aphasia remains insufficient for drawing definitive conclusions and making recommendations. Similarly, with regards to dysphagia post-stroke, Lefaucheur et al. (56) assert that due to the variability in results and protocols, it remains uncertain whether rTMS provides therapeutic benefits for patients experiencing persistent dysphagia in the post-acute or chronic stages of stroke. But, given that post-stroke dysphagia often exhibits rapid recovery, it is advised to administer rTMS in the early stages of the disease to maximize therapeutic benefits (56).

The use of rTMS as a treatment option for post-stroke aphasia and dysphagia shows potential but also poses challenges. While rTMS is a non-invasive approach with the potential to induce neuroplastic changes, making it a possibly effective intervention for both conditions, the current state of research reveals uncertainty. The heterogeneity in study methodologies, stimulation protocols, and outcome measures and the lack of consensus regarding optimal stimulation sites and ecological outcome measures make it difficult to synthesize evidence, develop standardized treatment guidelines and enhance the clinical relevance of findings. Also, the variability in tracking variables and controlling factors, particularly in post-stroke aphasia trials, highlights the importance of standardization and international collaboration to enable meaningful comparisons across diverse linguistic and cultural contexts. To improve the precision and applicability of future research, studies must clearly define target groups and include robust risk stratification based on key prognostic markers for aphasia and dysphagia, such as lesion size and location, overall health status, and baseline functional abilities. For instance, the predictive swallowing score (PRESS) model (57) uses easily measured predictors suitable for various clinical settings that, according to the researchers, can be further refined by adding instrumental biomarkers and advanced neuroimaging, improving decision-making, and promoting personalized medicine for patients that have suffered a stroke. Addressing all the aforementioned gaps and challenges, this non-invasive neuromodulation approach may become an effective add-on or standalone treatment for improving functional outcomes and QoL for individuals affected by stroke induced aphasia and/or dysphagia.

## Author contributions

AG: Writing – review & editing, Methodology, Conceptualization.
